# Symptom Profiles of a Convenience Sample of Patients with COVID-19 — United States, January–April 2020

**DOI:** 10.15585/mmwr.mm6928a2

**Published:** 2020-07-17

**Authors:** Rachel M. Burke, Marie E. Killerby, Suzanne Newton, Candace E. Ashworth, Abby L. Berns, Skyler Brennan, Jonathan M. Bressler, Erica Bye, Richard Crawford, Laurel Harduar Morano, Nathaniel M. Lewis, Tiffanie M. Markus, Jennifer S. Read, Tamara Rissman, Joanne Taylor, Jacqueline E. Tate, Claire M. Midgley, Case Investigation Form Working Group, Case Investigation Form Working Group, Neha Balachandran, Rebecca M. Dahl, Mary Dott, Zunera Gilani, Aaron Grober, Jessica Leung, Michelle O’Hegarty, John Person, Jessica N. Ricaldi, Nicole M. Roth, James J. Sejvar, Tom Shimabukuro, Cuc H. Tran, John T. Watson, Hilary Whitham, Howard Chiou, Paula Clogher, Lindsey M. Duca, Alissa Dratch, Amanda Feldpausch, Mary-Margaret Fill, Isaac Ghinai, Michelle Holshue, Sarah Scott, Ryan Westergaard

**Affiliations:** ^1^CDC COVID-19 Response Team; ^2^Virginia Department of Health; ^3^Rhode Island Department of Health; ^4^Georgia Department of Health; ^5^Alaska Department of Health and Social Services; ^6^Minnesota Department of Health; ^7^Preventive Medicine Residency Program, University of Wisconsin–Madison, Wisconsin; ^8^Pennsylvania Department of Health; ^9^Utah Department of Health; ^10^Epidemic Intelligence Service, CDC; ^11^Emerging Infections Program, Vanderbilt University Medical Center, Nashville, Tennessee; ^12^Vermont Department of Health and the Department of Pediatrics, University of Vermont, Burlington, Vermont; ^13^Connecticut Emerging Infections Program, Yale School of Public Health, New Haven, Connecticut.; CDC COVID-19 Response Team; CDC COVID-19 Response Team; CDC COVID-19 Response Team; CDC COVID-19 Response Team; CDC COVID-19 Response Team; CDC COVID-19 Response Team; CDC COVID-19 Response Team; CDC COVID-19 Response Team; CDC COVID-19 Response Team; CDC COVID-19 Response Team; CDC COVID-19 Response Team; CDC COVID-19 Response Team; CDC COVID-19 Response Team; CDC COVID-19 Response Team; CDC COVID-19 Response Team; COVID-19 Response Team; Connecticut Emerging Infections Program, Yale School of Public Health; COVID-19 Response Team; Orange County Healthcare Agency; Georgia Department of Public Health; Tennessee Department of Health; COVID-19 Response Team; COVID-19 Response Team; COVID-19 Response Team; Wisconsin Department of Health Services.

Coronavirus disease 2019 (COVID-19) was first detected in the United States in January 2020 (*1*), and by mid-July, approximately 3.4 million cases had been reported in the United States (*2*). Information about symptoms among U.S. COVID-19 patients is limited, especially among nonhospitalized patients. To better understand symptom profiles of patients with laboratory-confirmed COVID-19 in the United States, CDC used an optional questionnaire to collect detailed information on a convenience sample of COVID-19 patients from participating states. Symptom data were analyzed by age group, sex, hospitalization status, and symptom onset date relative to expansion of testing guidelines on March 8, 2020 (*3*). Among 164 symptomatic patients with known onset during January 14–April 4, 2020, a total of 158 (96%) reported fever, cough, or shortness of breath. Among 57 hospitalized adult patients (aged ≥18 years), 39 (68%) reported all three of these symptoms, compared with 25 (31%) of the 81 nonhospitalized adult patients. Gastrointestinal (GI) symptoms and other symptoms, such as chills, myalgia, headache, and fatigue, also were commonly reported, especially after expansion of testing guidelines. To aid prompt recognition of COVID-19, clinicians and public health professionals should be aware that COVID-19 can cause a wide variety of symptoms.

The CDC COVID-19 case investigation form is a detailed questionnaire that was completed as an optional activity by participating states* for a subset of laboratory-confirmed COVID-19 cases identified through care-seeking, surveillance, or contact tracing. This subset of cases was selected at the state level through convenience sampling, with guidance to include cases with a range of ages and severities. Staff members at local or state health departments or CDC personnel deployed to support health departments interviewed patients or their proxies and reviewed medical records to complete the case investigation form. The case investigation form was used to collect demographic, epidemiologic, and clinical information (including symptoms) about COVID-19 patients. Patients were asked about a series of commonly and less commonly reported symptoms, and then asked to report any additional symptoms. This investigation was determined by CDC to be public health surveillance. Therefore, approval by CDC’s Institutional Review Board was not required.

This analysis included only symptomatic persons. Symptom data for a given patient were considered sufficient for analysis if the date of symptom onset was included and if responses indicated the presence or absence of at least 50% of symptoms that were specifically asked about. For this report, fever (measured or subjective), cough, or shortness of breath, all of which have been frequently described among COVID-19 patients, were classified as typical signs or symptoms. GI symptoms included nausea, abdominal pain, vomiting, or diarrhea. Analysis was descriptive, and an absolute difference of ≥15 percentage points was considered notable. Because this was a convenience sample, no statistical tests were performed. Symptom profiles were examined by age, sex, and hospitalization status. Stratifications by age and hospitalization status are presented because this was a nonrepresentative sample. Patients were excluded from stratification by hospitalization status if their age or hospitalization status was unknown or if they were reported to be hospitalized for reasons other than illness severity, such as for public health isolation. Symptoms also were examined by date of onset relative to March 8, 2020, when CDC released a Health Alert Network (HAN) notification giving updated guidance that COVID-19 testing be performed based on clinical judgment, thus widening testing eligibility to include persons with milder illness or atypical symptoms (*3*). Cases also were categorized retrospectively by whether or not they would have met the clinical component of the case definition approved by the Council of State and Territorial Epidemiologists (CSTE) on April 5, 2020 (*4*). That definition requires meeting one or more of three sets of criteria: 1) cough, shortness of breath, or difficulty breathing; 2) at least two of the following symptoms: fever (measured or subjective), chills, rigors,^†^ myalgia, headache, sore throat, or new changes in smell or taste; or 3) severe respiratory illness with either clinical or radiographic evidence of pneumonia or acute respiratory distress syndrome, without an alternative more likely diagnosis.

Sixteen participating states^§^ submitted case investigation forms containing data collected during January 19–June 3, 2020, for 199 COVID-19 patients. Among those patients, 192 (97%) reported experiencing any symptoms, six (3%) reported experiencing no symptoms, and one (<1%) had unknown symptom status. Sufficient symptom data for analysis were available for 164 (85%) patients. Symptom onset ranged from January 14 to April 4. The median patient age was 50 years (range = 1 month–95 years), and 56% of patients were male. Among the sample of 147 (90%) patients for whom age and hospitalization status were known, 90 (61%) were not hospitalized, including nine (10%) aged <18 years and 81 (90%) aged ≥18 years. All of the 57 (39%) patients who were hospitalized for clinical management were aged ≥18 years. Each of the following symptoms was reported by >50% of patients: cough (84%), fever (80%), myalgia (63%), chills (63%), fatigue (62%), headache (59%), and shortness of breath (57%) (Figure). Approximately half of patients reported one or more GI symptoms; among these, diarrhea was reported most frequently (38%) and vomiting least frequently (13%). Among adult patients, shortness of breath was more commonly reported by hospitalized than by nonhospitalized patients (82% versus 38%). In contrast, new changes in smell and taste and rhinorrhea were reported by a higher percentage of nonhospitalized patients (22% and 51%, respectively) than hospitalized patients (7% and 21%, respectively).

**FIGURE:**
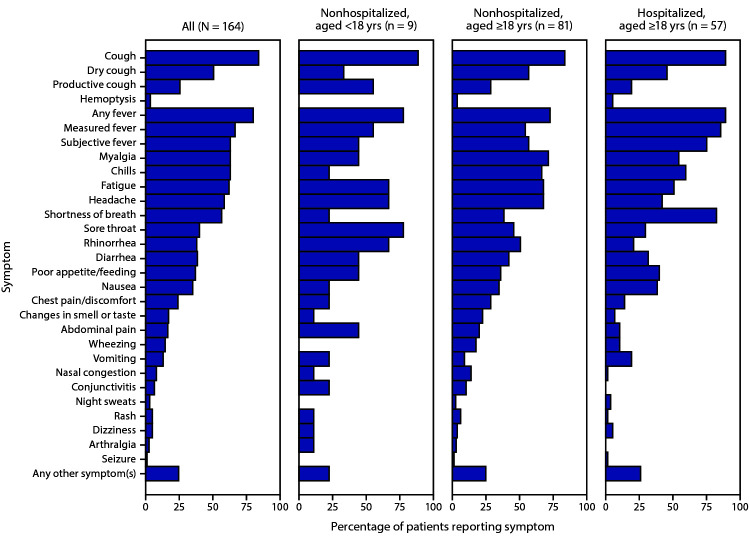
Reported symptoms among 164 patients with laboratory-confirmed COVID-19, by age and hospitalization status*^^,†,§ ^^— United States, January–April 2020 **Abbreviation:** COVID-19 = coronavirus disease 2019. * Seventeen persons with missing age or hospitalization status, or who were hospitalized for public health purposes (isolation), are included in the total but excluded from subgroups. ^†^ Chest pain or discomfort includes the solicited symptom “chest pain,” as well as free text key words “chest fullness,” “chest tightness,” “chest pressure,” or “chest discomfort.” ^§^ Symptoms of arthralgia, dizziness, nasal congestion, night sweats, and changes in smell or taste were captured from free text write-in.

Nearly all of the 164 symptomatic patients (96%) reported one or more of the typical signs and symptoms of fever, cough, or shortness of breath; 45% of patients reported all three (Table). Among all adults, the reported prevalence of all three signs and symptoms increased with increasing age; 23 of 61 (38%) persons aged 18–44 years, 24 of 50 (48%) persons aged 45–64 years, and 20 of 36 (56%) persons aged ≥65 years reported all three typical signs and symptoms. However, among the 57 hospitalized adults, 68% of whom reported all three symptoms, prevalence did not differ meaningfully by age or sex. Among 81 nonhospitalized adult patients, 25 (31%) reported all three symptoms. Among 97 patients who reported one or more GI symptoms, 93 (96%) also reported one or more typical symptom.

**TABLE:** Reported COVID-19 symptom profiles among a convenience sample of 164 patients with laboratory-confirmed COVID-19* — United States, January–April 2020

Symptom profile	No. (%)			
	Total	Nonhospitalized children (<18 yrs)	Nonhospitalized adults (≥18 yrs)	Hospitalized adults (≥18 yrs)
	(N = 164)	(n = 9)	(n = 81)	(n = 57)
**Typical symptoms^†^**				
No typical symptoms reported	6 (4)	1 (11)	4 (5)	0 (0)
At least one typical symptom reported	158 (96)	8 (89)	77 (95)	57 (100)
All three typical symptoms reported	74 (45)	2 (22)	25 (31)	39 (68)
**GI symptoms^§^**				
No GI symptoms reported	67 (41)	2 (22)	29 (36)	27 (47)
At least one GI symptom reported	97 (59)	7 (78)	52 (64)	30 (53)
Three or more GI symptoms reported	16 (10)	1 (11)	7 (9)	7 (12)
**Symptom combinations**				
Typical symptom(s) with GI symptom(s)	93 (57)	6 (67)	49 (60)	30 (53)
Typical symptom(s) without GI symptom(s)	65 (40)	2 (22)	28 (35)	27 (47)
GI symptom(s) without typical symptom(s)	4 (2)	1 (11)	3 (4)	0 (0)
No GI or typical symptom(s)	2 (1)	0 (0)	1 (1)	0 (0)

**Abbreviations:** COVID-19 = coronavirus 2019; GI = gastrointestinal.

* Total includes 17 persons with missing age or hospitalization status, or who were hospitalized for public health purposes (isolation). Those 17 persons are excluded from the subgroups.

^†^ Typical symptoms include fever (measured or subjective), cough, or shortness of breath.

^§^ GI symptoms include diarrhea, vomiting, nausea, or abdominal pain.

Among all 164 symptomatic patients, only four would not have met the clinical component of the CSTE case definition. Those four included an infant whose only reported signs were vomiting and increased irritability, and for whom health care was not sought. They also included a woman who reported only chills and a “tickle in her throat” (without cough), who was hospitalized for isolation and had a normal chest radiograph. A second woman reported only fever and did not seek health care. A third woman reported only rhinorrhea and congestion, “never felt sick,” and had only a telemedicine consultation. All four patients either had close contact with a patient with confirmed COVID-19 or had traveled to an area with sustained community transmission.

Symptoms reported during a period of broader testing eligibility might reflect a more complete COVID-19 symptom profile. Therefore, symptoms reported in patients who had illness onset before and after testing guidelines were expanded on March 8 were compared. Throughout the analysis period, patients commonly reported the typical signs and symptoms of fever, cough, and shortness of breath, but other symptoms were more commonly reported after March 8 compared with before March 8. For example, one or more signs or symptoms in the CSTE case definition (chills, myalgia, headache, sore throat, or new changes in smell or taste) were reported for 85% of the patients who had onset of symptoms before March 8, compared with 94% of patients with onset after March 8. Among patients with onset before March 8, 48% were reported to have one or more GI symptoms, compared with 68% of patients with onset after March 8. In particular, the percentage of patients reporting diarrhea increased from 20% before March 8 to 53% after that date. Changes in smell or taste were reported much more frequently after March 8 (30%) than before (3%). For cases with onset after March 8, olfactory or taste disorders were reported among 29% of nonhospitalized and 20% of hospitalized patients, and by 37% of females and 20% of males. Other symptoms reported more commonly after March 8 included rhinorrhea (20% before, 52% after), fatigue (42% before, 77% after), and chest pain or discomfort (8% before, 35% after). After testing expanded, a greater proportion of patients for whom reports were submitted were not hospitalized, but the increased reporting of symptoms other than fever, cough, or shortness of breath was observed in hospitalized and nonhospitalized patients.

## Discussion

Among 164 symptomatic COVID-19 patients, nearly all experienced fever, cough, or shortness of breath, and all but four would have met the CSTE clinical case definition. However, a wide variety of other symptoms were also reported; chills, myalgia, headache, fatigue, and the presence of at least one GI symptom (most commonly diarrhea) were each reported by >50% of patients. The occurrence of these symptoms in patients with COVID-19 has also been reported elsewhere (*5*–*7*). Symptoms other than fever, cough, and shortness of breath were reported more commonly after testing guidelines were expanded. This change might reflect an expansion of the types of patients eligible for testing and an increased awareness of other COVID-19 symptoms over time, such as changes in smell or taste. Few differences in symptom profile were notable by age or sex, especially when stratifying by hospitalization status; however, hospitalized patients (many of whom were older) more frequently reported experiencing fever, cough, and shortness of breath. As reported by others, changes in smell or taste were more commonly reported by women than by men (*6*,*8*).

The findings in this report are subject to at least five limitations. First, these cases represent a convenience sample of predominantly symptomatic COVID-19 patients reported by 16 states during a timeframe that included a period when testing was restricted to certain patients. For this reason, results are not generalizable. For instance, hospitalized patients are likely overrepresented as a result of the guidance to sample cases with a “range of severities” and because early in the outbreak, testing was limited to more severe cases. This sampling strategy also precludes estimation of the prevalence of asymptomatic infection because only symptomatic patients are systematically detected as part of standard public health activities. Second, because case investigation forms were occasionally completed by proxy or several weeks after illness onset, some symptoms were unknown or might have been forgotten. Third, case investigation forms completed soon after illness onset might not have captured symptoms that developed later. Fourth, the prevalence of unsolicited (“write-in”) symptoms might be underestimated; this might have been most likely in the early phases of the U.S. outbreak, when less was known about the possible spectrum of symptoms. Conversely, the prevalence of fever, cough, and shortness of breath might have been overestimated, even after expansion of testing guidelines, because widespread awareness of these symptoms might have affected testing practices. Finally, sample sizes were small, particularly for children, limiting the ability to draw conclusions about differences by age group.

Clinicians and public health professionals should be aware that COVID-19 can manifest a range of symptoms. Because prompt identification of COVID-19 patients is important to slow the spread of disease, testing should be considered for patients experiencing 1) fever, cough, or shortness of breath; 2) symptoms included in the CSTE case definition, including chills, myalgia, or headache; 3) other symptoms, including diarrhea or fatigue, especially if reported along with fever, cough, or shortness of breath; and 4) for asymptomatic persons, based on clinical or public health judgment (*9*). Representative symptom data from U.S. patients across the spectrum of COVID-19 illness severity, including data on the timing of symptom development, are needed to inform clinical case definitions and guidance for symptom screening or testing criteria.

**Summary**

**What is already known about this topic?**

Information about COVID-19 symptoms, especially among nonhospitalized U.S. patients, is limited and not well characterized across the spectrum of illness severity.

**What is added by this report?**

Fever, cough, or shortness of breath were commonly reported among a convenience sample of U.S. COVID-19 patients with symptom onset during January–April and a range of illness severity; gastrointestinal symptoms and other symptoms, such as chills, myalgia, headache, and fatigue, also were commonly reported.

**What are the implications for public health practice?**

U.S. COVID-19 patients report a wide range of symptoms across a spectrum of illness severity; these findings can inform clinical case definitions or testing guidance to aid prompt recognition to slow the spread of COVID-19.

## Footnotes

* States that participated in this supplemental activity include Alaska, Arizona, California, Connecticut, Georgia, Hawaii, Illinois, Minnesota, Pennsylvania, Rhode Island, Tennessee, Utah, Virginia, Vermont, Washington, and Wisconsin.
